# A likelihood ratio test for the homogeneity of between-study variance in network meta-analysis

**DOI:** 10.1186/s13643-021-01859-3

**Published:** 2021-12-09

**Authors:** Dapeng Hu, Chong Wang, Annette M. O’Connor

**Affiliations:** 1 Department of Statistics, College of Liberal Arts and Sciences, Iowa State University, Ames, IA USA; 2 Department of Veterinary Diagnostic and Production Animal Medicine, College of Veterinary Medicine, Iowa State University, Ames, IA USA; 3 Professor of Epidemiology, Chairperson of the Department of Large Animal Clinical Sciences, College of Veterinary Medicine, Michigan State University, East Lansing, MI USA

**Keywords:** Heterogeneity, Between-study variance, Network meta-analysis, Hypothesis testing

## Abstract

**Background:**

Network meta-analysis (NMA) is a statistical method used to combine results from several clinical trials and simultaneously compare multiple treatments using direct and indirect evidence. Statistical heterogeneity is a characteristic describing the variability in the intervention effects being evaluated in the different studies in network meta-analysis. One approach to dealing with statistical heterogeneity is to perform a random effects network meta-analysis that incorporates a between-study variance into the statistical model. A common assumption in the random effects model for network meta-analysis is the homogeneity of between-study variance across all interventions. However, there are applications of NMA where the single between-study assumption is potentially incorrect and instead the model should incorporate more than one between-study variances.

**Methods:**

In this paper, we develop an approach to testing the homogeneity of between-study variance assumption based on a likelihood ratio test. A simulation study was conducted to assess the type I error and power of the proposed test. This method is then applied to a network meta-analysis of antibiotic treatments for Bovine respiratory disease (BRD).

**Results:**

The type I error rate was well controlled in the Monte Carlo simulation. We found statistical evidence (*p* value = 0.052) against the homogeneous between-study variance assumption in the network meta-analysis BRD. The point estimate and confidence interval of relative effect sizes are strongly influenced by this assumption.

**Conclusions:**

Since homogeneous between-study variance assumption is a strong assumption, it is crucial to test the validity of this assumption before conducting a network meta-analysis. Here we propose and validate a method for testing this single between-study variance assumption which is widely used for many NMA.

## Background

Network meta-analysis (NMA) is an approach to combining evidence from multiple studies of multiple interventions and obtaining estimates of all possible intervention comparisons using indirect and direct evidence. Common approaches to network meta-analysis include a fixed effect model and a random effects model. The random effects model assumes that the true effect size can differ from study to study, because the effect size in each study is derived from a random distribution of effect sizes. Several assumptions about the data generating mechanism are made in network meta-analysis. Common to the fixed effect model and random effects model is the exchangeability assumption. The exchangeability assumption relates to the study populations and states that the randomized trials are similar on average, in all important factors other than the intervention comparison being made [1, 2]. The exchangeability assumption implies the consistency condition is valid [3], i.e., the relative effect of A to B, can be derived from the difference using data from C compared to A and C compared to B for any treatments A, B, and C. A commonly used assumption unique to the random effects model is a single between-study variation for all treatments [4]. Assuming that all effects sizes across all treatments have the same between-study variation is a strong assumption. However, there are applications of NMA where the single between-study assumption is potentially incorrect and instead the model should incorporate more than one between-study variance estimate. A few approaches have been proposed to allow different between-study variation across treatment comparisons. Lu (2009) proposed a Bayesian approach to modeling between-study variance structures under the consistency assumption [5]. White (2012) proposed a partially structured heterogeneity model that allows for two between-study variances but did not have a practical reason for doing so [6]. Although these approaches have been proposed, the single between-study variation assumption remains widely used. In practice, there is a lack of guidance for when the homogeneous assumption should be challenged. The decision to assume one or more between-study variance should be informed primarily by the reviewers’ knowledge of the data generating mechanism. However, the results from statistical testing, comparison of results of the NMA under both assumptions and the magnitude of variance estimates can also support any decisions made about the structure of between-study variance.

Recently we conducted several network meta-analyses of interventions to prevent bovine respiratory disease in feedlot cattle, where the assumption of a single between-study variance was questionable based on our knowledge of the biology of the disease and interventions included in the meta-analysis. Turner et al. [7] found heterogeneity might be related to the type of comparison and models with heterogeneous variances have been proposed with different informative priors under the Bayesian framework [8]. However, this is not applicable in frequentist framework. Additionally, limited work has been reported on testing the assumption of a single between-study variance across all treatment comparisons. Therefore, the objective of this project was not to model the between-study variance structure, but to develop an approach to testing the homogeneity of between-study variance in a network meta-analysis based on the likelihood method. For network meta-analysis, several different methods of calculating the single between-study variance have been proposed [9–11]. However, we were unable to identify any commonly used approaches to testing this assumption compared to an alternative that two or more between-study variances exist based on a characteristic of the underlying studies. The sequence of the paper is as follows:

- Section 2: The motivating example
- Section 3: The model and proposed likelihood ratio test (LRT)
- Section 4: The evaluation of the LRT using two methods
- Section 5: Discussion of the evaluation and application.

## Motivating example

The motivating example involved bovine respiratory disease, a multi-agent disease of cattle. Bovine respiratory disease (BRD) is the most economically important disease of feedlot cattle and therefore knowledge of the comparative efficacy of interventions to prevent, control and treat BRD is critically important. One common approach to preventing bovine respiratory disease is to administer antibiotics to all cattle at arrival at the feedlot. The aim of administering antibiotics at arrival is to preemptively treat animals with sub-clinical BRD and to prevent BRD in animals at risk. Trials conducted to assess how effective antibiotics are for this purpose, use the proportion of treated animals detected with BRD after a period of time, usually 28 days, as the outcome. The data available for assessing the comparative efficacy of antibiotics for this purpose included comparisons of antibiotic to antibiotic, and comparisons of an antibiotic to no-treatment. For BRD prevention, the assumption of a single between-study effect for both types of comparisons is biologically questionable. It is known that some antibiotics are highly effective at treating and preventing BRD because the mechanism of action is very broad spectrum. An example of such a group of antibiotics is the macrolide group. Antibiotics in this group have consistent high quality evidence of low BRD risk after 28 days when administered at arrival [12, 13]. This means that trials that compare a macrolide to a macrolide would be expected to have a comparative effect size near zero, if the effect size is measured as the log odds ratio (log OR). The between-study variation of macrolide to macrolide trials is therefore expected to be small. However, for trials that compare a board spectrum antibiotic, such as a macrolide, to a non-treated control, the expected variation in the effect size is much larger, because the risk of BRD in the 1st 28 days in cattle is highly variable in non-treated cattle. The data suggests that some groups of untreated cattle have close to zero animals detected with BRD after 28 days while other groups have 50% or more animals with BRD. The result of this naturally expected variation in BRD risk in the 1st 28 days of feedlotting in non-treated animals is a wider variation in the comparative effect sizes when active drugs such as macrolides are compared to non-treated groups. For example, if the macrolide is highly effective, we expect that the number of animals treated for BRD after 28 days will be close to zero regardless of the underlying risk of BRD in the group. However, the non treated group may have anywhere from zero to 100%. When these data are converted to a distribution of the comparative effect sizes (log OR), it is natural that more variation is expected between these active to no-treatment trials than the trials that are macrolide to macrolide. There are several other scenarios in BRD, where the assumption of a single between-study variance for all comparisons is questionable. For example, to prevent BRD in animals arriving at the feedlot, antibiotics or vaccines might be used. As with a no-treatment group, the response to vaccination is highly variable, yet the response to broad spectrum antibiotics like, macrolides is highly consistent. Therefore in a network of evidence that compared the efficacy of antibiotic and vaccines to prevent BRD, we would naturally expect the vaccine to vaccine comparisons to be more variable than board spectrum antibiotic to broad spectrum antibiotic comparison. It is these examples, that motivated the work described below.

## Methods

### The likelihood for a random effects model of network meta-analysis under consistency assumption

This section provides the basic model form used for formulating the likelihood ratio test. In the following, we consider *T* treatments that are compared in *I* studies each with *n*_*i*_ arms. The set of treatments included in study *i* is given by *T*_*i*_. Let ***y***_*i*_ denote the estimates of relative effects for the *i*th study, $\phantom {\dot {i}\!}\boldsymbol {y}_{i} = (y_{i,1},..., y_{i,n_{i}-1})^{T}$ and ***y***=(***y***_1_,...,***y***_*T*_). The study specific treatment effects of study *i* are given by ***θ***_*i*_ where ***θ***=(***θ***_*i*_,...,***θ***_*I*_). Then we have

$$ \boldsymbol{y}_{i} = \boldsymbol{\theta}_{i} + \boldsymbol{\epsilon}_{i}. $$

where ***ε***_*i*_ represents the vector of errors of study *i*. ***ε***_*i*_ is assumed to be normally distributed and independent across studies and its covariance is cov(***ε***_*i*_) = *S*_*i*_. *S*_*i*_ is a diagonal matrix of size (*n*_*i*_−1)×(*n*_*i*_−1) and is a scalar if study *i* only has two arms. The distribution of ***y*** is

$$ \boldsymbol{y} \sim \text{MVN}\left(\boldsymbol{\theta}, \boldsymbol{S}\right), $$

where ***S*** is a block diagonal matrix with each block ***S***_*i*_,*i*=1,...,*I*. As the consistency assumption is made in the random effects model, all treatment effects are uniquely determined by *T*−1 basic treatment comparisons with a common reference (usually a placebo). These basic parameters are denoted by the vector ***d***. The relative effect size of all other possible treatment comparisons in the network are called functional parameters which can be obtained from the basic parameters. For example, if *d*_1,2_ and *d*_1,3_ are basic parameters in the network, then *d*_2,3_, a functional parameter, can be obtained by

$$ d_{2,3} = d_{1,3} - d_{1,2}. $$

Let ***X*** denote the design matrix of size *I*×(*T*−1). Each row of ***X*** corresponds to one study specific comparison and the columns represent the basic comparisons and. 1, 0, and -1 are the possible values in the design matrix. If one row of ***X*** only has one element of 1 and other elements are 0, then this study specific comparison is a basic comparison. If 1 and -1 occur in one row, then the relative effect parameter of the corresponding comparison is a functional parameter. For each study *i*, the design matrix is denoted by ***X***_*i*_. Then,

$$ \boldsymbol{\theta}_{i} = \boldsymbol{X}_{i} \boldsymbol{d} + \boldsymbol{\delta}_{i}, $$

where ***δ***_*i*_ is the vector of between–study heterogeneity of study *i*. The random effects model usually assume ***δ***_*i*_ to be normally distributed. If study *i* only has two arms, then ***δ***_*i*_∼N(0,*τ*^2^), otherwise, ***δ***_*i*_∼MVN(0,***V***_*i*_), where the values of the diagonal elements of ***V***_*i*_ are *τ*^2^ and off–diagonal values are *τ*^2^/2 [5, 14]. The values of the off–diagonal elements are determined by the assumption that every source of direct evidence has the same between-study variance. The distribution of ***θ*** is

$$ \boldsymbol{\theta} \sim \text{MVN}(\boldsymbol{X d}, \boldsymbol{V}), $$

where *V* is a block diagonal matrix with each block *V*_*i*_,*i*=1,...,*I*. The between-study heterogeneity is assumed to be independent of within-study errors. Hence, the marginal distribution of ***y*** is

$$ \boldsymbol{y} \sim \text{MVN}(\boldsymbol{X d}, \boldsymbol{S} + \boldsymbol{V}). $$

If we know *τ*^2^, then the maximum likelihood estimate of ***d*** is

$$ \hat{\boldsymbol{d}} = (\boldsymbol{X}^{T} (\boldsymbol{S} + \boldsymbol{V})^{-1} \boldsymbol{X})^{-1} \boldsymbol{X}^{T} (\boldsymbol{S} + \boldsymbol{V})^{-1} \boldsymbol{y}. $$

### Likelihood ratio test for the between-study variance parameter

Here we discuss an approach to testing the assumption of a single *τ*^2^. Based on our motivating example, the between-study variance parameter for non–active to active treatment comparisons and active to active treatment comparisons are denoted by $\tau _{n}^{2}$ and $\tau _{a}^{2}$ respectively. The hypotheses to be tested are

$$ \mathrm{H}_{0}: \tau_{n}^{2} = \tau_{a}^{2} = \tau^{2}, \quad \mathrm{H}_{a}: \tau_{n}^{2} \neq \tau_{a}^{2}. $$

The log-likelihood function under the null hypothesis is

$$\begin{aligned} \text{lnL}(\boldsymbol{d}, \tau^{2}) =& -\frac{1}{2} \text{log} |\boldsymbol{S} + \boldsymbol{V} | - \frac{1}{2} (\boldsymbol{y} - \boldsymbol{X d})^{'} (\boldsymbol{S} + \boldsymbol{V})^{-1} (\boldsymbol{y} - \boldsymbol{X d}) \\&- \frac{I}{2}\text{log}(2\pi)\\ =& -\frac{1}{2} \sum_{i=1}^{I} \text{log} |(\boldsymbol{S}_{i} + \boldsymbol{V}_{i})| - \frac{1}{2} (\boldsymbol{y} - \boldsymbol{X d})^{'} (\boldsymbol{S} + \boldsymbol{V})^{-1} (\boldsymbol{y} - \boldsymbol{X d}) \\&- \frac{I}{2}\text{log}(2\pi). \end{aligned} $$

Under the null hypothesis, the structure of ***V***_*i*_ is discussed in section 3. There are two potential data forms for ***V***_*i*_ under the alternative hypothesis. If study *i* only contains active treatments, then the values of diagonal elements of ***V***_*i*_ are $\tau _{a}^{2}$ and off-diagonal values are $\tau _{a}^{2}/2$. If non-active controls are included in study *i*, then the diagonal values (variance) are $\tau _{n}^{2}$ and the off-diagonal values (co-variance) are $\tau _{n}^{2} - \tau _{a}^{2}/2$.

For example, suppose study *i* is a three-arm trial that compares a non-active control (denoted by *N*) with two active treatments (denoted by *A*_1_,*A*_2_). The between-study variance-covariance matrix for study *i* is

$$\text{Var}\left(\left[\begin{array}{c} \theta_{i, NA_{1}}\\ \theta_{i, NA_{2}} \end{array}\right] \right) = \left[\begin{array}{cc} \tau^{2}_{n} & \tau^{2}_{n} - \frac{1}{2}\tau^{2}_{a}\\ \tau^{2}_{n} - \frac{1}{2}\tau^{2}_{a} & \tau^{2}_{n} \\ \end{array}\right]. $$

Since $\phantom {\dot {i}\!}\text {Var}(\theta _{i, N A_{2}} - \theta _{i, N A_{1}}) = \text {Var}(\theta _{i, N A_{2}}) + \text {Var}(\theta _{i, N A_{1}}) - 2\text {Cov}(\theta _{i, N A_{2}}, \theta _{i, N A_{1}})$, the covariance (off-diagonal) is given by

$$\begin{aligned} \text{Cov}(\theta_{i, N A_{2}}, \theta_{i, N A_{1}}) =& \left(\text{Var}(\theta_{i, N A_{2}}) + \text{Var}(\theta_{i, N A_{1}})\right. \\&\left.- \text{Var}(\theta_{i, N A_{2}} - \theta_{i, N A_{1}})\right)/2 \\ =& \left(\text{Var}(\theta_{i, N A_{2}}) + \text{Var}(\theta_{i, N A_{1}}) \right.\\&\left.- \text{Var}(\theta_{i, A_{1} A_{2}})\right)/2\\ =& \left(\tau^{2}_{n} + \tau^{2}_{n} - \tau^{2}_{a} \right)/2\\ =& \tau^{2}_{n} - \frac{1}{2}\tau^{2}_{a}. \end{aligned} $$

To make the variance-covariance matrix semi-positive definite, the covariance should follow the following inequality:

$$|\text{Cov}(\theta_{i, N A_{2}}, \theta_{i, N A_{1}})| \leq \sqrt{\text{Var}(\theta_{i, N A_{2}}) \text{Var}(\theta_{i, N A_{1}})}. $$

To meet this inequality the following constrains are placed on $\tau ^{2}_{n}$ and $\tau ^{2}_{a}$:

$$\begin{array}{*{20}l} -\tau^{2}_{n} \leq \tau^{2}_{n} - \frac{1}{2}\tau^{2}_{a} \leq \tau^{2}_{n} \Longleftrightarrow 0 \leq \tau^{2}_{a} \leq 4\tau^{2}_{n}. \end{array} $$

Here a three-arm trial is used to illustrate the covariance matrix structure and the constrains. Similar structures and the same constrain are applicable to trials with more than three arms. The likelihood ratio test (LRT) statistic is

$$-2\left(\text{lnL} \left(\hat{\boldsymbol{d}}, \hat{\tau}^{2}\right) - \text{lnL} \left(\hat{\boldsymbol{d}}, \hat{\tau}_{n}^{2}, \hat{\tau}_{a}^{2}\right) \right), $$

where the estimates of the parameters are the maximum likelihood estimates. The asymptotic distribution of this test statistic is $\chi _{1}^{2}$. Given $\hat {\tau }^{2}$, the maximum likelihood estimate of $\boldsymbol {\hat {d}}$ is

$$ \boldsymbol{\hat{d}} = (\boldsymbol{X}^{T} \left(\hat{\boldsymbol{S}} + \hat{\boldsymbol{V}}\right)^{-1} \boldsymbol{X})^{-1} \boldsymbol{X}^{T} \left(\hat{\boldsymbol{S}} + \hat{\boldsymbol{V}}\right)^{-1} \boldsymbol{y}. $$

## Real data implementation and simulation results

**Fig. 1:**
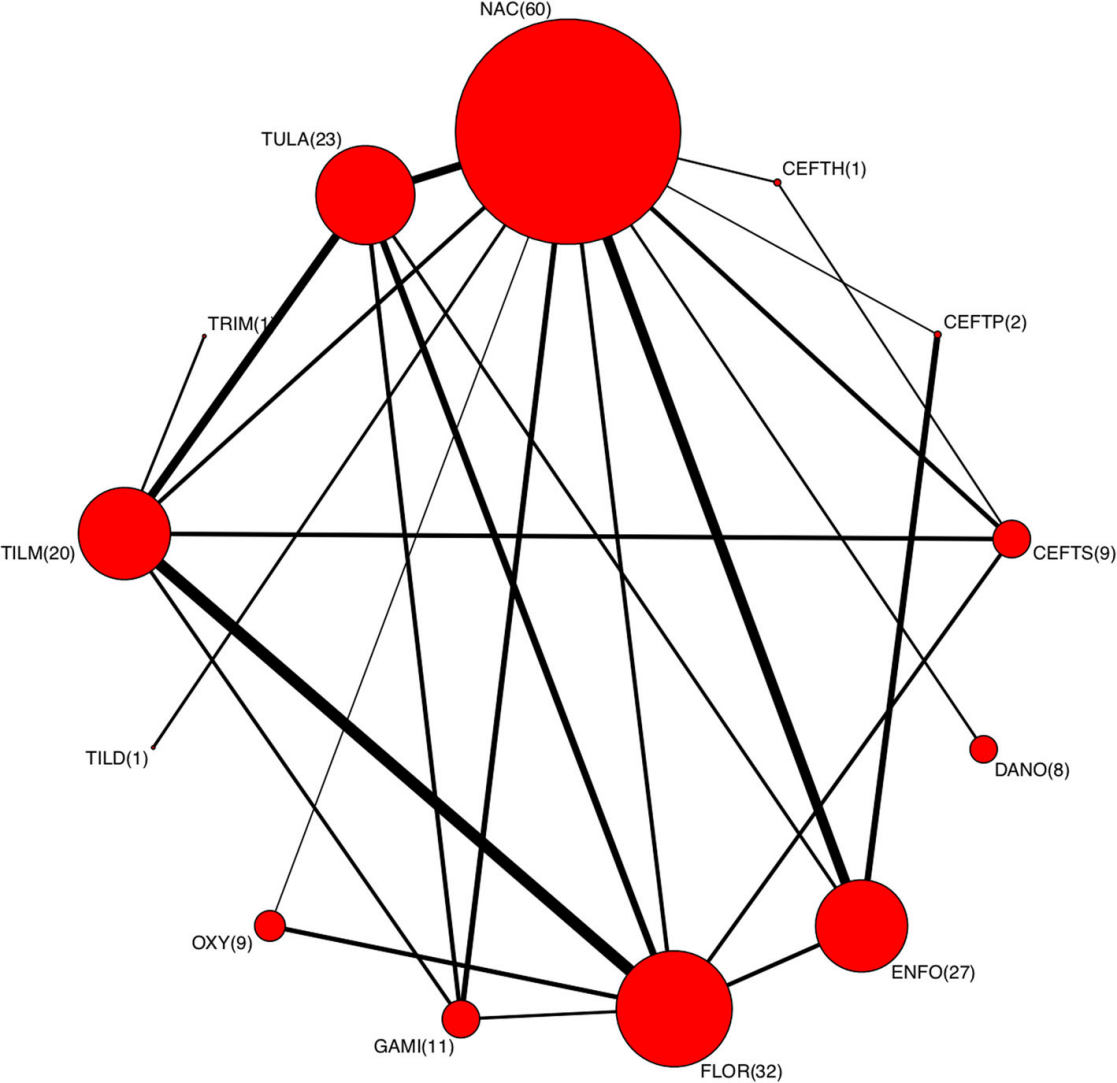
The network plot of the treatment arms for bovine respiratory disease in feedlot cattle. The size of the node represents the magnitude of the number of arms and the thickness of the edges represents the total size of direct comparisons between each treatment pair

The data used are from a network meta–analysis of antibiotic treatments for BRD in feedlot cattle [15]. The evidence network consists of 204 trial arms from 98 studies. Eight of the 98 trials have three arms. The total number of participants in all studies is 26,132, with the number of participants in a study ranging between 34 and 1726. Among the total 26,132 participants, 9467 had the event. There are 13 treatments in the network: non-active control (NAC), ceftiofur hydrochloride (CEFTH), ceftiofur bollus in pinna (CEFTP), ceftiofur sodium (CEFTS), danofloxacin (DANO), enrofloxacin (ENFO), florfenicol (FLOR), gamithromycin (GAMI), oxytetracycle (OXY) used at multiple doses, tildipirosin (TILD), tilmicosin (TILM), trimethoprim (TRIM), and tulathromycin (TULA). The outcome is the log odds ratio of the proportion of treated animals detected with BRD. A negative log OR means treatment benefit for the numerator treatment compared to the referent. The within–study variance is obtained using delta method. For example, in a 2–arm study with reported number of events *r*_1_ and *r*_2_ and sample sizes *N*_1_ and *N*_2_, the within-study variance is calculated by 1/*r*_1_+1/(*N*_1_−*r*_1_)+1/*r*_2_+1/(*N*_2_−*r*_2_). The number of pairwise comparisons is 106 in total with 66 non-active control to active treatments (N2A) comparisons and 40 active to active treatments (A2A) comparisons. The network plot is shown in Fig. 1. The size of the node is proportional to the number of arms and the thickness of the edges represents the total size of direct comparisons between each treatment pair. The number in the parentheses after a treatment abbreviation is the number of studies containing that treatment. The absence of a line means that there is no direct comparison in the evidence network.

To evaluate the performance of the proposed LRT, two methods have been used. The first method is based on the asymptotic distribution (*χ*^2^) of the LRT statistic and the second method is established on the Monte Carlo simulation. Maximum likelihood estimation is applied to obtain the basic effect size parameters and *τ*^2^ under the null and alternative hypothesis. We simulated 1000 data sets under the null hypothesis being true (a single between–study variance for all treatment comparisons) to assess the type I error rate and another 1000 data sets where the alternative hypothesis was true (two between-study variance, one for N2A and one for A2A) to evaluate the power given the significance level of 0.05. Under the null hypothesis, the simulated data $\boldsymbol {y}_{H_{0}}$ is generated from the real data ***y*** by

$$\boldsymbol{y}_{H_{0}} = \text{MVN}(\boldsymbol{X}\hat{\boldsymbol{d}}_{H_{0}},\hat{S}+\hat{V}_{H_{0}}), $$

where $\hat {\boldsymbol {d}}_{H_{0}}$ is the maximum likelihood estimate given $\hat {\tau }^{2}$. Since the LRT statistic under the null hypothesis follows a chi square distribution when the sample size goes to infinity, we also assessed the type I and power for the scenario where the number of studies is five times the original to determine if the type I error can be well controlled when the sample size per comparison is larger. This increased-size dataset has the same network structure as the real data. For example, in the original network, there is no study comparing treatment TRIM with NAC, and this is also the case in the simulated network. Only one study compares TRIM with TILM as shown in Fig. 1, whereas for the increased-size data set there are five studies simulated for this comparison.

### Assessing type I error rate and power of the test based on the chi square distribution

For each simulated dataset where the null hypothesis was true (a single between–study variance for all treatment comparisons), the maximum likelihood estimates were obtained and the LRT statistic calculated. The proportion of these 1000 LRTs that are beyond the 95% quantile of the $\chi _{1}^{2}$ distribution is the estimated type I error rate. The power can be obtained by applying the same procedure on each simulated dataset where the alternative hypothesis is true (two between-study variance, one for N2A and one for A2A).

### Assessing type I error and power of the likelihood test based on the Monte Carlo simulation

An alternative approach to the chi-square approach is a simulation based approach to testing. This procedure is as follows:

1. For each simulated dataset where the null hypothesis is true, the maximum likelihood estimates are obtained under both hypotheses and LRT is calculated, denoted by LRT_*b*_(*b*∈{1,...,1000}).
2. One thousand data sets are generated given the estimates in this simulated dataset under the null hypothesis. We used the MLE to obtain parameter estimates under both hypotheses and calculate LRT statistics, denoted by LRT_*b,m*_*m*∈{1,...,1000}
3. The *p* value of the LRT_*b*_ is $\frac {1}{1000}\sum _{m=1}^{1000} \text {LRT}_{b,m} > \text {LRT}_{b}$, denoted by *p*_*b*_.
4. The proportion of rejection is the type I error which is obtained by $\frac {1}{1000}\sum _{b=1}^{1000} I_{p_{b}<0.05}$, where *I* is the identity function.

For estimating power, the only change is to use each simulated dataset under the alternative hypothesis being true in the step 1.

### Results

The values of *τ*^2^ observed in the original BRD dataset are shown in Table 1. The *p* value of the likelihood ratio test based on the asymptotic distribution of the test statistic is 0.028 indicating a significant difference between the two heterogeneity parameters but the type I error rate inflates in this case. The simulation-based *p* value is very close to 0.05. Hence, making decision only relies on the cut-off of 0.05 for the *p* value of the LRT is not convincing. The heterogeneity parameters values estimated under two models are meaningfully different. The estimated between-study variance for the non-active control to active treatments comparison is four times larger than that for active to active treatments. This difference would have an impact on the confidence intervals of the relative effects of the comparisons in the network, especially for comparisons with fewer studies. Then the Wald 95% confidence interval of $\hat {\tau }^{2}_{n} - \hat {\tau }^{2}_{a}$ is calculated and given by (0.0282,0.8469) which indicates a significant difference from 0.

**Table 1:** Estimates of *τ*^2^ from the analysis of the a meta-analysis network for bovine respiratory disease treatments using maximum likelihood estimation

Number of studies (N2A, A2A)	*P* value under $\chi _{1}^{2}$	Monte Carlo *P*-value	*τ*^2^ under *H*_0_	*τ*^2^ under *H*_*a*_ ($\tau _{n}^{2}, \tau _{a}^{2}$)
(66, 40)	0.028	0.052	0.3096	(0.5659, 0.1283)

**Fig. 2:**
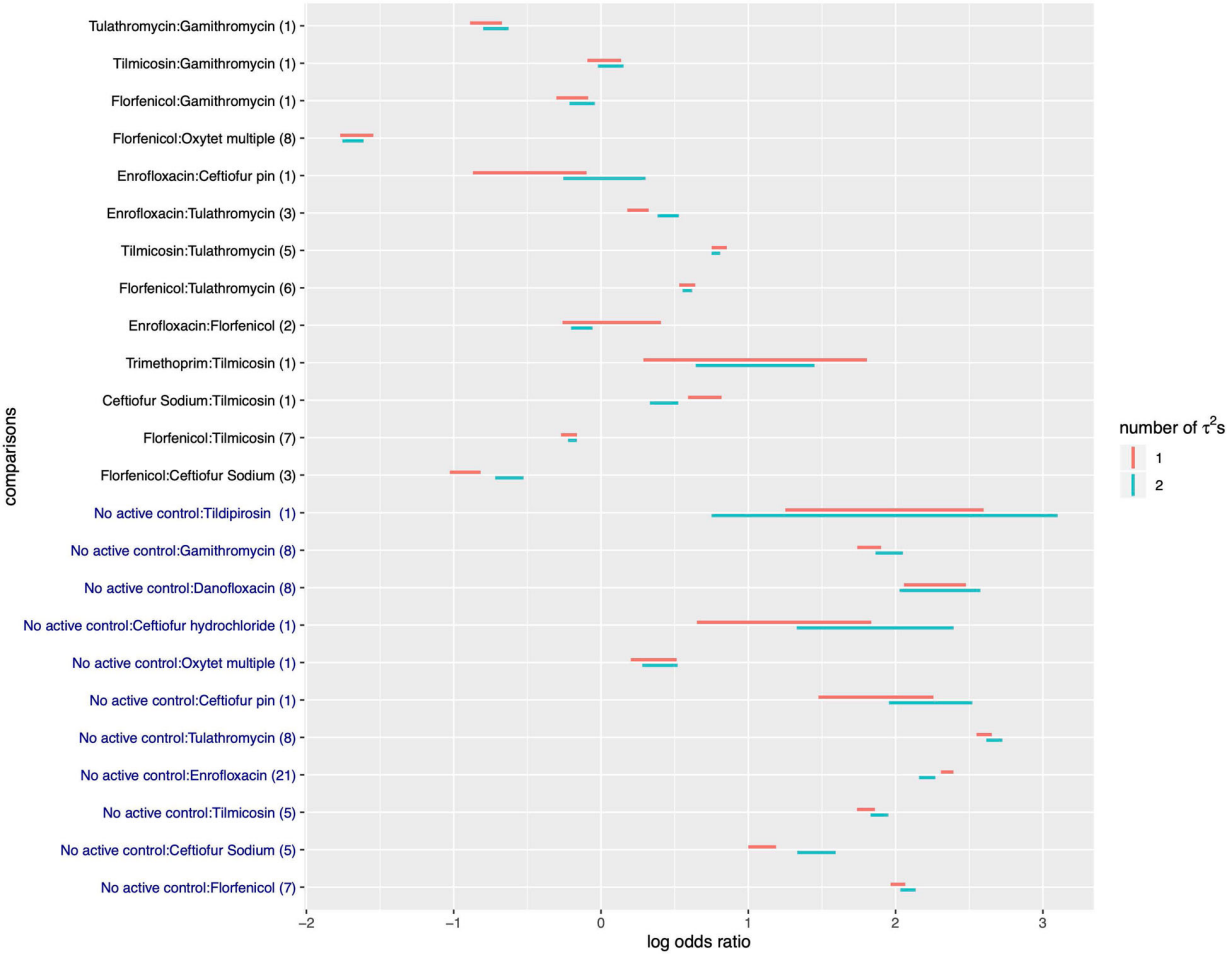
The approximate 95% confidence intervals of the log odds ratios of the treatment comparisons presented in the network plot under the models with one and two heterogeneity parameters. The comparisons on the *y*-axis in blue are non-active control to active treatment comparisons. Those in black are active to active comparisons

The effect of models with different heterogeneity parameters on the point estimates and confidence intervals of the relative effect sizes, are presented in Fig. 2. Figure 2 shows the 95% confidence intervals of the log odds ratios of the treatment pairs presented in the network plot under the models with one and two between-study variance parameters. Treatment comparisons that involve only one study which has small study size tends to have wider confidence interval because of the large within-study variance. It can be seen in Fig. 2 that some confidence intervals change markedly in width under the different models. Some of the point estimates of the relative effect sizes shift because of the change of estimates in between-study variances which would vary the weight of direct and indirect comparisons. The estimate of *τ*^2^ of N2A comparison in two *τ*^2^s model is greater than that in one *τ*^2^ model and the *τ*^2^ of A2A comparison is opposite. Therefore, the width of confidence intervals tends to be narrower for A2A comparisons in the two *τ*^2^ model than in the one *τ*^2^ model. Also, most of the point estimates of the effect sizes of N2A comparisons shift to the right under the two *τ*^2^ model. It is not easy to predict the direction of the change of the point estimate of effect size or the width of the confidence interval in the two *τ*^2^ model for each comparison since it is a mixed weight change of direct and indirect comparisons.

The results of the study the likelihood ratio test performance in Table 2 shows the type I error rate and the power analysis results. The simulation based on the original data is labeled (60, 40) to indicated the number of studies. While the increased size data is labeled (330, 200). The increased-size data set have the same network structure as the real data. For the asymptotic distribution of the test statistics, the type I error is above 5%, i.e., 8.3%. Increasing the number of studies reduced the type I error drop to 5%, i.e., 4.4%. While in the Monte Carlo simulation-based evaluation, the type I errors are controlled in both settings. The power was suitable for all methods and datasets. By combining the results in Table 2 with those in Table 1, we can say there is a significant difference between the heterogeneity parameter of non-active control to active treatments comparisons and of active to active treatments comparisons. In practice, if the *p* value of the LRT statistic is very close to the cut-off (i.e., 0.05 in this paper) like in this example, depending on *p* value only to make decision is not conclusive. Visual inspection of the results from the two models and how these results differ is helpful in reaching a conclusion.

**Table 2:** Results of assessment of type I error and power for two approaches to testing the homogeneity of between-study variance

Number of studies (N2A, A2A)	Evaluation method	Type I error	Power
(66, 40)	Monte Carlo simulation	4.8%	88.9%
(66, 40)	*χ*^2^	8.3%	93.5%
(330, 200)	Monte Carlo simulation	4.4%	100%
(330, 200)	*χ*^2^	5%	100%

The values in the parentheses are the number of comparisons of N2A and A2A type, respectively

## Conclusions

We have proposed a likelihood ratio test for testing the homogeneity of the between-study variance parameter for the random effect network meta-analysis model. We illustrate this method with an example for testing the homogeneity between the non-active control to active treatments comparisons and of active to active treatments comparisons. Our example applied this likelihood ratio test in a network meta-analyses which contained a non-active control (or placebo or no-treatment) and our understanding of the biology of this example, raised concerns about the single between-study variance esti- mate. There are many other situations that this method can be applied, for example, the between–study heterogeneity for a pharmacological treatment vs surgery comparison might be different from that of a comparison of two pharmacological treatments. We also developed the variance-covariance matrix structure of the model with two heterogeneity variance parameters. In the motivating example, we applied the test and found the significant difference of the between-study variance of two types of comparisons. We have explored two ways to define the *p* value based on the same LRT statistic, one using the asymptotic *χ*^2^ distribution and the other using a Monte Carlo simulated sampling distribution. In practice, we would recommend using the Monte Carlo *p* value, which has a better control of the type I error, especially when the number of studies is limited. The estimation method for the basic parameters and between-study variance is MLE. There are many literature comparing different methods of estimating the between-study variance parameter[16–19]. Different estimators may have different distributions and our method is based on the MLE. That is not to say MLE is the best estimator but we just propose a possibility that the between-study variance may not be the same across all comparisons and we use MLE and likelihood ratio test to show the single heterogeneity parameter assumption may not hold in some cases. Tests for this assumption using other estimators are possible extensions. Our likelihood ratio test is developed based on a model where the consistency condition is considered valid. If the consistency condition is not met, alternative models can be used to address inconsistency and the likelihood ratio test can be developed under the new model in an analogous fashion. Testing the homogeneity of between-study variance in network meta-analysis with inconsistency is an interesting topic that we leave as a possible future work.

## Data Availability

We provide the R code and data we used in this paper in https://github.com/dapengh/test_the_heterogeneity_of_the_between-study_variance.

## Abbreviations

NMA: Network meta-analysis
BRD: Bovine respiratory disease
LRT: Likelihood ratio test
OR: Odds ratio
A2A: Active to active
N2A: Non-active to active
NAC: Non-active control
CEFTH: Ceftiofur hydrochloride
CEFTP: Ceftiofur bollus in pinna
CEFTS: Ceftiofur sodium
DANO: Danofloxacin
ENFO: Enrofloxacin
FLOR: Florfenicol
GAMI: Gamithromycin
OXY: Oxytetracycle
TILD: Tildipirosin
TILM: Tilmicosin
TRIM: Trimethoprim
TULA: Tulathromycin
